# Yeast as a Tool to Understand the Significance of Human Disease-Associated Gene Variants

**DOI:** 10.3390/genes12091303

**Published:** 2021-08-24

**Authors:** Tiziana Cervelli, Alvaro Galli

**Affiliations:** Yeast Genetics and Genomics Group, Laboratory of Functional Genetics and Genomics, Institute of Clinical Physiology CNR, Via Moruzzi 1, 56125 Pisa, Italy; tizicerv@ifc.cnr.it

**Keywords:** yeast, *Saccharomyces cerevisiae*, functional assays, Mendelian disease, cancer, human gene variants

## Abstract

At present, the great challenge in human genetics is to provide significance to the growing amount of human disease-associated gene variants identified by next generation DNA sequencing technologies. Increasing evidences suggest that model organisms are of pivotal importance to addressing this issue. Due to its genetic tractability, the yeast *Saccharomyces cerevisiae* represents a valuable model organism for understanding human genetic variability. In the present review, we show how *S. cerevisiae* has been used to study variants of genes involved in different diseases and in different pathways, highlighting the versatility of this model organism.

## 1. Introduction

The advent of next generation DNA sequences technologies allows us to identify individual genetic variants, and this revolutionized the strategy for discovering the cause of several genetic and multifactorial diseases. The association between genetic variants and disease risk can potentially overturn our understanding of common disease. The discovery of pathways and processes that are causally involved in a disease, so far not linked, may open the way towards the development of targeted therapies and prevention strategies. However, the knowledge of individual genetic variants is not sufficient for determining the causality or predisposition to a particular disease without a clear picture of the impact of the genetic variants on the protein activity. The analysis of genetic variants resulted in the successful identification of impaired proteins underlying many Mendelian disorders. However, our knowledge is far from complete, especially for rare variants [1]. While for genes involved in monogenic diseases the association of the variant with the disease is applicable, such association is often not possible in the case of polygenic disorders. Therefore, forming precise predictions for the most common diseases is still an ambitious challenge. In order to fill the gap between the identification of the variant and its classification, several approaches are necessary. One approach is based on the use of algorithms that predict the impact of amino acid substitution on the structure and, hence, on the functionality of the protein [2]. Even though computational methods are considered a valid tool in clinical practice for interpreting genetic variants [3], they have limited predictive power [4,5,6]. This approach is not sufficient for explaining the complexity of human diseases because it focuses only on the protein without considering pathways and interactions inside the cell. The other approach is based on the use of model organisms (MOs) such as yeast, worm, fly, zebrafish, and mouse. Indeed, the International Rare Disease Research Consortium (IRDiRC) recognizes that MOs constitute a powerful experimental system for investigating the impact of gene variants on the protein activity, for determining their biological function, and for identifing potential therapies [7]. 

The yeast *Saccharomyces cerevisiae* is a unicellular eukaryote representing an invaluable tool for uncovering much of the basic biology considering that it has helped to characterize pathways common to human cells. An example is the discovery and mechanistic understanding of autophagy, a fundamental process involved in cancer, neurological disorders, and other diseases, allowing Yoshinori Ohsumi to be awarded with the Nobel Prize in Physiology in 2016 [8]. The advantage of using yeast is that is an amenable organism, easy to manipulate, and for which many genetic tools have been created, such as the yeast deletion pool that enables genome-wide genetic and chemical screenings [9]. Numerous examples of how studies in model organisms have informed human disease, in some cases resulting in the development of a treatment, are nicely described in a recently published review [10]. 

We have previously shown the contribution of yeast in the interpretation of variants of genes involved in DNA repair [11]. In the present review, we show how yeast research can be integral to unraveling disease-associated human genetic variants of genes coding for proteins involved in different pathways, and how the study in yeast alongside the analysis in patient cells has been used to underpin the significance of genetic variants. 

## 2. Principles of Yeast Humanization

The study of a human gene in *S. cerevisiae*, generally called yeast humanization, is extensively reviewed in [12,13]. The type of humanization depends on the gene under investigation. If the human gene has no ortholog in yeast, the only approach possible is the expression of the human gene in yeast as a readout for functional studies. In order to evaluate the impact of gene variants, the simplest condition is that the human gene expression causes a growth inhibition per sé in the wild type (wt) strain. Such condition enables evaluating the loss of function of the variant by the growth recovery. Examples of human genes without orthologs causing growth arrest are BAX [14], PARP1 [15], BRCA1 [16], p53 [17], and α-SYNUCLEIN [18]. More challenging is the case where the expression of the human gene has no effect on growth in the wt strain; therefore, an ad hoc yeast mutant (single or multiple mutants) highlighting a phenotype that can be leveraged needs to be created. The oncogene BRAF has been reported not to have an ortholog in yeast, and BRAF overexpression does not affect the growth of yeast wt strain [19]. If the human gene has an ortholog, the functional assay can be performed in the strain deleted for the yeast ortholog or mutating the yeast gene in the nucleotide corresponding to that of the human gene [20]. If the yeast ortholog gene is essential, the construction of the strain carrying the deleted or mutated gene has to be performed in heterozygosis; this is accomplished by mutating one allele of a diploid strain or expressing the wild type gene from a plasmid in a haploid strain. Later on, the yeast strain is transformed with the plasmid for the expression of the human gene and then, through a specific selection, the yeast wt orthologous gene is lost. In particular, the major issue is to have a measurable phenotype. If the yeast counterpart is an essential gene, the effect of human gene expression in yeast is the rescue of lethal growth defects. In the case of non-essential genes, most of them causing minimal growth defects when disrupted, the discernible phenotype has to be identified in mutant strains or in assays under restrictive media conditions, such as the following: growth in non-fermentable carbon source for genes affecting mitochondrial functions [21], absence of metabolite for gene involved in metabolic pathway [21,22], adding chemicals to sensitize the yeast strain for genes involved in DNA repair [23,24], or converting the non-essential yeast gene to an essential gene by disrupting a synthetic lethal partner [25].

## 3. The Study of Genetic Variants by High Throughput Approaches

**Figure 1:**
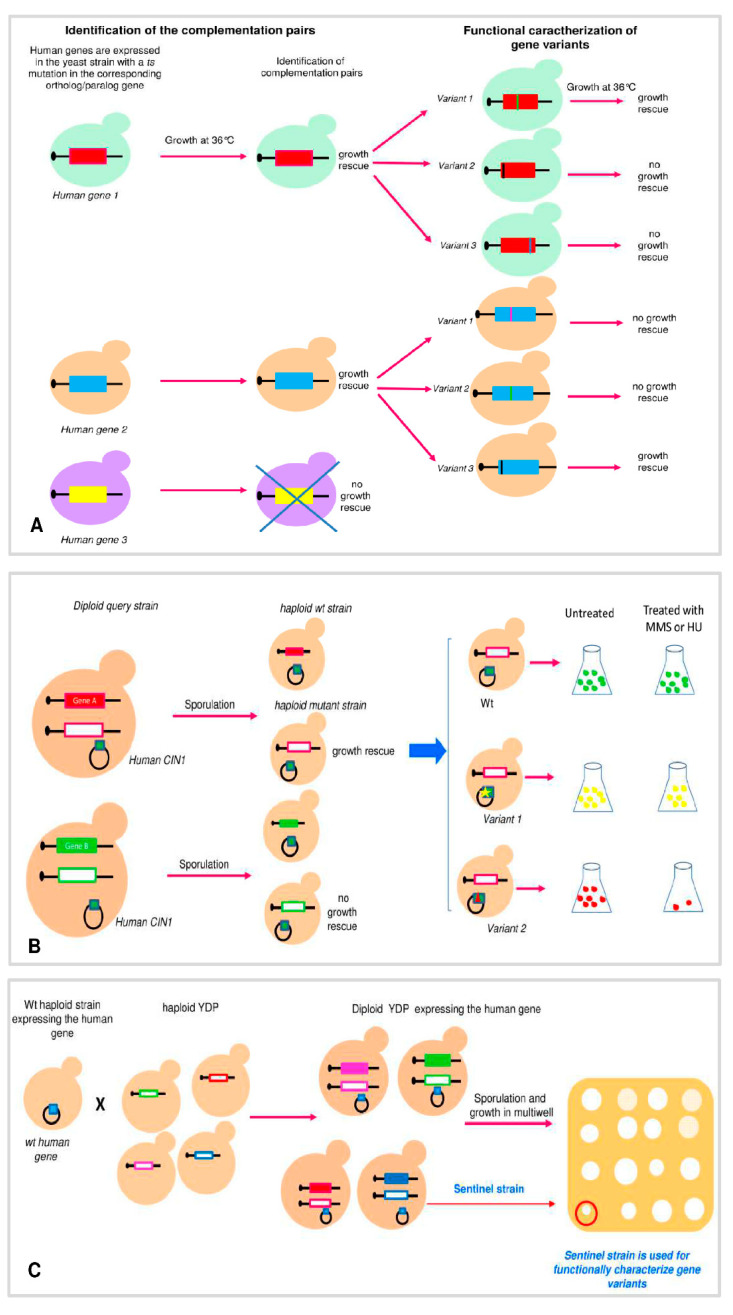
Schematic representation of high throughput approach. (**A**) The wild type (wt) human genes associated with a disease, with an essential yeast ortholog/paralog, have been expressed in yeast temperature sensitivity (ts) mutant strains and grown at restrictive temperature to test their ability to rescue the ts phenotype. After the identification of the complementation pair, missense variants of the selected human gene were assayed. (**B**) Large scale study for the functional characterization of variants of human chromosome instability (CIN) genes. *Diploid query strain*: Strain carrying the plasmid for the overexpression of the human gene and heterozygosis for a CIN gene. Sporulation of a query strain produces *haploid yeast strains* expressing a human CIN. Growth will be observed in strains expressing a human CIN complementing the yeast mutant. Gene variants were expressed in the identified strain and grown in the presence of DNA damaging agents for the classification. (**C**) sentinel interaction mapping (SIM) approach. The wt *haploid strains* expressing the human gene are crossed with haploid yeast deletion pool (YDP) strains. Sporulation of the resulting diploid strains produces haploid strains. Those showing growth defects in the presence of the human gene are the sentinel strains that were used to classify the gene variants.

In high throughput studies, several genes involved in human diseases are taken into consideration (Figure 1). The aim of Sun et al. [26] was to develop a platform for classifying pathogenic human variants of as many genes as possible. The principle of the platform is the ability to complement the function of a yeast gene with a human wt gene or with its variants. Human genes selected for the study have a yeast homologous gene that, when mutated, confers a temperature sensitivity (ts) phenotype. Mutant ts strains show normal growth at permissive temperature (30 °C) but are unable to grow at non-permissive temperature (36 °C). Each human wt gene, expressed via a yeast promoter, was transformed into the corresponding yeast ts strain; later on, yeast cells were grown at the non-permissive temperature to determine the ability of the gene to complement the ts phenotype. Once identified, the complementation pair and disease-associated missense variants were transformed in the ts yeast strain in order to evaluate the rescue of growth at the non-permissive temperature (Figure 1A). The effect of their expression was determined by growth on solid media or in liquid media by spectrophotometric time courses. Functional assay data, a robust statistical analysis, and comparison with predictive tools led the authors to the assignment of a functional score to 145 variants belonging to genes such as the Guanosine Diphosphate Dissociation Inhibitor1 (*HsGDI*) associated with Non-Syndromic X-Linked Intellectual Disability; phosphoglycerate kinase (*HsPGK1*) associated with phosphoglycerate kinase deficiency; and thiamine pyrophosphokinase (*HsTPK1*) associated with the thiamine pyrophosphokinase deficiency syndrome 5 [26].

The same group extended the functional characterization to human genes having one or more essential yeast paralogs for which a ts mutant was available (Figure 1A). Paralogs are genes arising from the duplication of a gene and, generally, have a distinct specialized function from the original gene. They identified 34 human–yeast paralog complementation pairs of which 33 (97%) were novel. Successively, they assessed the ability of human/yeast paralog complementation assays to identify the pathogenic variants of a subset of human genes. By this approach, they confirmed the association with Smith–McCort dysplasia of two variants of the gene *RAB33B*, which encodes a small GTP-binding protein of the RAB family [27]. These findings are in agreement with the two algorithms, PolyPhen-2 (http://genetics.bwh.harvard.edu/pph2) and PROVEAN (http://genetics.bwh.harvard.edu/pph2), which predicted them to be deleterious. The results obtained with these two approaches showed that yeast-based functional complementation assays reached three times the sensitivity of computational methods for detecting disease variants at the same high threshold of precision. Moreover, the results of this paper highlighted that having a single protein domain in common may be sufficient for complementation, whereas sequence similarity and closer evolutionary relationships are not good predictors of complementation relationships [28,29].

Hamza et al. [29] performed a large-scale study for genes involved in chromosome instability (CIN) (Figure 1B). The screening was performed by transforming plasmids for the expression of human genes in a pool of heterozygous diploid strains carrying one allele with deletion in a yeast essential CIN. After transformation of human genes, either with single plasmids or pool of plasmids, sporulation (formation of haploid cells) was induced. Haploid cells (spores) that are able to grow indicate that the expressed human gene complements the growth defect of yeast essential CIN (complementation pair). Finally, they selected three yeast CIN genes, corresponding to four complementation pairs (yCDC9:*hLIG1*, yPOB3:*hSSRP1*, yGLC7:*hPPP1CA*, and yGLC7:*hPPP1CC*), and assessed the impact of single amino acid substitutions in the human protein identified by cBIOPORTAL (https://www.cbioportal.org/) and COSMIC (https://cancer.sanger.ac.uk/cosmic). A measurable phenotype for mutants of CIN genes is the sensitivity to DNA damaging agents such as methyl methane sulfonate (MMS) and hydroxyurea (HU). Most of yeast strain expressing the selected gene variants grew in a complete medium, such as those expressing the wt genes; therefore, the functional assay was performed by assessing the ability of variants to rescue growth of the null mutant in the absence or in the presence of MMS and HU. Human gene variants unable to complement the sensitivity to MMS or HU of null mutant were considered pathogenic. Contrary to Yang et al. [28], they observed that prediction is not always in agreement with the functional test underscoring the importance of functional characterization of protein variants [29].

A large-scale study has evaluated the pathogenicity of human gene variants by a modified version of synthetic genetic array analysis (SGA). Genetic interaction occurs when the combination of two mutations results in an unpredictable phenotype as result of genes that buffer one another or affect the same pathway. Yeast SGA analysis offers an efficient approach for the systematic construction of double mutants and allows a global analysis of synthetic genetic interactions. In a typical SGA screen, a haploid yeast strain with a query mutation is crossed to the ~4800 haploid strains of the yeast deletion pool (YDP) such that meiotic progeny harboring both mutations can be scored for fitness defects by determining colony-size measurements. In synthetic dosage lethality (SDL) screening, a variant of SG and a query mutation is crossed into yeast deletion strains, each of which overexpresses a different gene [30,31]. This technology has great potential since it is applicable to a wide variety of human disease genes without requiring detailed knowledge of its biology, and it is scalable, thus allowing rapid classification of hundreds of variants. Young et al. [32] called their approach sentinel interaction mapping (SIM) (Figure 1C). The first step of this approach is the identification, by SDL, of candidate haploid strains showing a phenotype upon the overexpression of the human gene (sentinel strains). SDL was performed by mating a wt haploid yeast strain overexpressing the gene of interest with strains of haploid YDP, followed by sporulation and induction of the expression of the human gene. The effect of the human gene on growth of the haploid deletion strain was evaluated in multi-well plates, and the results were processed by the program “Balony”, a freely available software designed for the extraction of quantitative data from array-based genetic screens [33]. Haploid strains showing growth defect in the presence of the human gene are the sentinel strains. Confirmed sentinel strains are then used for high-throughput liquid growth assays of multiple variants. As an example, they applied the technique to the human gene *PTEN*. They identified eight sentinel strains in which they studied the effect of variants by both agar plate-based arrays of yeast and microtiter-plate liquid growth assays. Interestingly, they found that different sentinel strains largely coincided on identifying the loss of function of variants. Moreover, the assay was highly informative also in the case that the human genes showed different phenotypes in the sentinels because it provided information on different aspects of the protein function. In a recent study, the functional characterization of *PTEN* variants by SIM was supported by a deep phenotypic profiling approach consisting of 18 functional assays in four other model systems, spanning diverse cellular environments ranging from molecular function to neuronal morphogenesis and behavior. In such a manner, the authors analyzed 106 missense and nonsense variants of *PTEN* [34].

## 4. The Study of Genetic Variation of Single Genes

**Figure 2:**
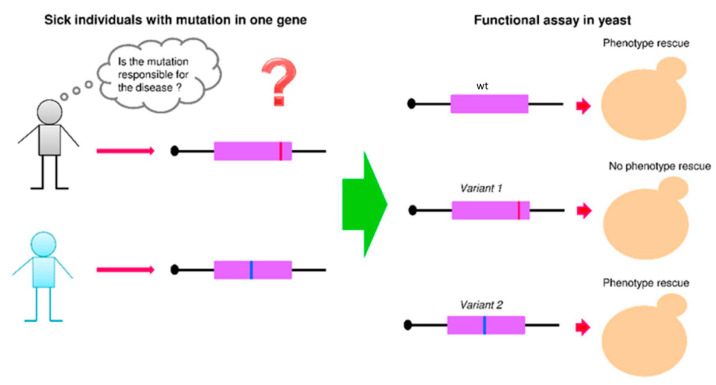
Schematic representation of the strategy for the characterization of variants identified in a patient. The wt and gene variants are expressed in the identified yeast strain for functional assays. Gene variants that do not rescue the phenotype are considered loss of function.

In addition to large-scale analysis of variants of several genes, there are studies focused on a single gene (Figure 2). Among studies on variants of a single gene, we can distinguish Mendelian diseases, rare diseases caused by germline mutations in one gene, and multifactorial diseases, such as cancer, where the predisposing mutations can be germline or somatic, and thus found in the tumor.

### 4.1. Yeast Functional Assay for Genes Involved in Mendelian Diseases

#### 4.1.1. Complementation Assays of Human Genes Coding for Enzymes or Subunits of Enzymes

Nowadays, a strategy to provide a name for a rare disease manifesting a phenotype common to several diseases, such as neurodevelopment disorders, is whole exome sequencing. However, sequencing is not enough to associate gene variants with diseases, and further studies to interpret the significance of the variants are needed. A strategy adopted by several groups is the use of *S. cerevisiae* to obtain information on the impact of the mutation on the activity of the protein. In these cases, the type of functional assay used is determined by the function of the protein (Table 1). 

When the human gene has an ortholog in yeast, the functional assay can be determined in strains deleted for the yeast counterpart of the human gene, as performed for the gene cystathionine β-synthase (*CBS*). Mutations in this gene cause homocystinuria, constituting a group of inborn error metabolism disorders characterized by an accumulation of the amino acid homocysteine in the serum and in the urine [35]. The enzyme Cbs converts homocysteine to cystathionine, regulating the amount of homocysteine inside the cells. Cysteine is not only an amino acid but also the precursor of glutathione, the major endogenous intracellular antioxidant. *CBS* has an ortholog in the yeast *S. cerevisiae*, the gene *CYS4* [36]. *CYS4* is an example of yeast ortholog quite different from the human counterpart because it lacks the heme binding domain, and thus may differ in details of its biochemical regulation. Regardless of that, complementation assays in yeast were useful for *CBS* variants classification. The yeast strain *cys4∆* is not able to grow in minimal medium but only in medium containing cysteine or glutathione and a little amount of vitamin B6, a defect that is complemented by human *CBS* (Table 2) [37]. These observations prompted the development of a yeast-based assay for the characterization of 84 missense mutations in different domains of Cbs protein. Therefore, the human *CBS* major allele, wild type in protein function, and 84 variants were expressed in the yeast strain *cys4∆*. Forty-six out of eighty-four alleles required glutathione addition to support growth, indicating severe loss of function. Surprisingly, 38 *CBS* alleles, found in patients with clinically significant homocysteinemia, were capable of supporting growth on medium lacking glutathione, and hence they seemed to retain substantial function according to this assay. Not convinced of neutrality of the 38 variants, the authors further analyzed them by using two quantitative assays consisting of growth in liquid medium at varying concentrations of vitamin B6 and expression in the *heme1∆* strain, followed by quantification of metabolites by LC–MS. By the three assays, they highlighted different phenotypes associated with the *CBS* alleles concluding that, for example, heme deficiencies could complicate the diagnosis and treatment of homocystinuria [38].

Another enzyme linked to an inborn error of metabolism is *PSAT1* (phosphoserine aminotransferase). A defect in serine biosynthesis causes a set of rare, autosomal recessive diseases known as the Neu-Laxova syndrome 2 (NLS2) or serine-deficiency disorders [39]. Similarly to *CBS*, *PSAT1* has a yeast ortholog, which is the gene *SER1*; thus, in order to test *PSAT1* variants, a complementation assay in the *ser1∆* strain was carried out. The mutant *ser1∆* is not able to grow in the absence of exogenously supplied serine; therefore, the quantitative readout of enzyme function was colony growth on minimal medium lacking serine (Table 2). The strategy adopted is a little different from that developed for *CBS1*: they constructed a strain where the yeast *SER1* gene was replaced with codon optimized human *PSAT1*. Strains containing *PSAT1* mutants, collected from the gnomAD (https://gnomad.broadinstitute.org/) and ClinVar3 (https://www.ncbi.nlm.nih.gov/clinvar/) database, were created and grown in medium without serine. This assay demonstrated a range of activity of the variants that allows the authors their classification [40]. 

The classification of variants identified in the human gene *SOD1* encoding superoxide dismutase-1, a major cytoplasmic antioxidant enzyme associated with 20% of cases of familial amyotrophic lateral sclerosis (fALS), is performed by mutagenizing the yeast ortholog. With this yeast model, they demonstrated that the mutant *SOD1* can disturb the amino acid biosynthesis and mediate cellular death, accounting for the neural degeneration in ALS (Table 2) [41].

If the deletion of the yeast ortholog does not present a measurable phenotype, double or triple mutants may have to be created, as in the case of adenine phosphoribosyl transferase (*APRT*), an enzyme involved in the salvage pathway of the purine metabolism. Purine metabolism plays an important role in nucleic acid synthesis, energy-dependent reactions, and cellular signaling. The interest towards this gene arises from the fact that mutations in *APRT* determined adenine phosphoribosyl transferase deficiency (APRTD), a rare monogenic disease [42]. This gene has more orthologs and, to mimic the loss of adenine observed in human cells, it is necessary to use the yeast triple mutant *ade2∆ apt1∆ aah1∆* since it is defective for de novo purine synthesis (called APRT-free) and not able to grow without additional purine (Table 2). In order to identify a key residue in *hAPRT* that affects the catalytic efficiencies of the enzyme, *hAPRT* wt and two variants, Y105F and E107L, were expressed in yeast *ade2∆ apt1∆ aah1∆* and grown on various purine-containing media. The assay confirmed that both APRT variants displayed a reduced growth rate in the absence of exogenous adenine, compared with the wild-type, in agreement with the in vitro analysis [43].

A yeast functional assay was also developed to study variants of unknown significance (VUS) of trio kinase (TKFC), a bifunctional enzyme component of the fructose metabolism pathway associated with a fatal infantile multisystem disorder [44]. The impacts of VUS, identified in two patients of two unrelated families, were first studied in cultured skin fibroblasts from patients. Unfortunately, the presence of VUS did not show a robust phenotype in fibroblasts; therefore, the authors explored the possibility for characterizing them in yeast. *TKFC* has two orthologs, *DAK1* and *DAK2*; thus, the functional assay was developed in the double mutant. TKFC could complement the function of the two yeast enzymes since, when overexpressed in the double mutant *dak1∆ dak2∆,* it restores the ability to grow in dihydroxyacetone (DHA) (Table 2). On the other hand, yeast strains carrying the *TKFC* VUS of the affected individuals were not able to grow on DHA as a carbon source. This result directly demonstrated the biochemical effect of the variants on TKFC/DAK function from which the authors could infer their likely pathogenicity [45].

In the previously described assays, the yeast ortholog was not an essential gene. Many reports demonstrate that is possible to use yeast to also study genes that are essential. This is the case of aminoacyl-tRNA transferases. Mutations in the gene methionyl-tRNA synthetase (*MARS1*) identified in Charcot-Marie-Tooth disorder type 2U (CMT2U); in glycyl-tRNA synthetase 1 (*GARS1*) associated with CMT (CMT2D) and with Spinal muscular atrophy; and infantile, James type (SMAJI), and valyl-tRNA synthetase (*VARS1*), associated with developmental encephalopathy with microcephaly have been successfully studied in yeast [46,47,48]. In these studies, gene variants are not retrieved from databases but identified in patients and their families because they are so rare that they have been never reported elsewhere. The three genes, *MARS1*, *GARS1*, and *VARS1*, complement the function of strains deleted for the yeast orthologs, *mes1∆*, *grs1∆*, and *vas1∆*, respectively (Table 2). The essentiality of these genes imposes a specific strategy for the study of the human counterpart. A wt yeast gene is expressed from a plasmid containing *URA3* as the selection marker allowing the construction of the strain with the deletion of the essential *ARS*. This strain is then transformed with a plasmid for the expression of the human *ARS* gene. Later on, in order to lose the plasmid for the expression of yeast *ARS,* yeast cells are grown in the presence of 5-Fluoroorotic acid (5-FOA). The 5-FOA acid is toxic for *URA3* positive cells because the enzyme coded by *URA3* gene will convert it into a toxic compound causing cell death. Interestingly, Siekiersk et al. demonstrated that *VARS1* variants are loss of function in yeast and precisely recapitulated some of the key neurological disease traits in Zebrafish [48]. This assay has been used for several *MARS1* variants and other aminoacyl transferase genes [49,50]. 

Similar strategies have been adopted to study the variants of *POLR2A* coding for RNA polymerase II subunit A gene with its yeast ortholog being an essential gene. Although the centrality of RNA polymerase II complex (pol II) in RNA biology is undisputed (it is responsible for transcription of about 21,000 human protein-encoding genes), *POLR2A* was not involved in human disease until 2016, when somatic mutations were discovered in a subset of meningioma [51]. Recently, *de novo* variants in *POLR2A* were identified in 16 cases of neurodevelopmental syndromes characterized by profound infantile-onset hypotonia and associated with the disease [52]. The impact of mutations on protein function was determined by several approaches such as mass spectrometry, expression in HeLa cells and yeast. The yeast ortholog, *RPB1*, is an essential gene; therefore, to study *POLR2A* variants in yeast, they used the strategy described above. Gene variants were then studied by constructing yeast strains carrying the yeast *RPB1* mutagenized in order to create the amino acid substitution observed in the human gene. In addition to the growth assay in *rpb1∆* strain, the *POLR2A* variants where further studied in yeast deleted in transcription factors *DST1* (yeast ortholog of *TFIIS*) or *SUB1* (yeast ortholog of *PC4*). This assay allowed determining how mutations in this subunit of RNA polymerase affect transcription fidelity. Altogether, on the basis of the results of both predictive and functional analyses, eleven variants were classified as probably disease-causing [52]. Recently, the yeast model for *POLR2A* variant classification was leveraged to study other cases of individuals with neurodevelopmental syndromes, strengthening the association of *POLR2A* with this disease [53].

A different approach has been used to study mutations in *NADSYN1*, coding for NAD synthetase 1 which is the final enzyme of the nicotinamide adenine dinucleotide (NAD) *de novo* synthesis pathway associated with congenital NAD deficiency disorder [54]. NAD is an essential metabolite that cannot be imported and functions as a coenzyme in many fundamental cellular redox reactions [55,56]. The yeast orthologous protein of Nadsyn1 is the essential protein Qns1, which converts the precursor nicotinic acid adenine dinucleotide (NaAD) to NAD [57]. The lethality of *QNS1* deletion may be overcome through exogenous supplementation of nicotinamide riboside (NR) (Table 2). In a case control study, six missense variants in the gene *NADSYN1* were identified in four unrelated families. In order to determine the pathogenicity of the variants, the authors utilized the predictive algorithm, PolyPhen-2, and exploited yeast to determine the functional impact. *NADSYN1*, expressed in the *qns1∆* strain, complement the Qns1 function; thus, the strain is able to grow in medium without NR. Thanks to this assay, the authors demonstrated that one variant out of six does not complement Qns1, suggesting a defect in the catalytic activity. Through the combination of yeast complementation assays with other assays such as the expression level of mutant protein and enzymatic analysis of mammalian-expressed NADSYN1 protein, they showed that disease-causal variants impair the catalytic activity of the enzyme, resulting in impaired NAD synthesis [58].

Yeast can be also used to determine the significance of variants identified in genes that have no ortholog as in the case of *PYROXD1*. *PYROXD1* encodes a protein with an N-terminal pyridine nucleotide-disulfide oxidoreductase domain and a C-terminal NADH-dependent nitrite reductase domain. Recently, biallelic *PYROXD1* gene mutations were identified by exome sequencing in nine patients with congenital myopathy from five unrelated families. The mutations segregated with the disorder in the families, suggesting that they are responsible for the disease. Unfortunately, at the time of variants’ discovery, the function of the protein was not determined. In order to study the activity of the protein and the effect of variants on protein activity, O’Grady et al. developed a complementation assay in yeast. They observed that the human PYROXD1 cDNA rescues the oxidative stress defect observed in yeast glutathione oxidoreductase *glr1∆* deletion mutant strain, indicating that PYROXD1 has an oxidoreductase activity in vivo in yeast cells. PYROXD1 variants expressed in *glr1∆* grown in medium containing H_2_O_2_ had a lower growth rate in the presence of H_2_O_2_ compared to wild-type PYROXD1 (Table 2). This was the evidence suggesting that newly identified patient mutations impair the oxidoreductase activity of PYROXD1 and that impaired redox regulation may be a primary cause of congenital muscle disease [59,60].

#### 4.1.2. Complementation Assays of Human Genes Coding for Transporters or Carriers

The TRAPP family proteins act as a trafficking complex and participate in events upstream of vesicle fusion and in some aspects of vesicle tethering to their correct intracellular target membrane. Recent evidence highlights the critical importance of the TRAPP complex to a spectrum of human diseases, now collectively termed TRAPPopathies [61]. Among the protein of the complex, TRAPPC2L [62] and TRAPPC4 [63] have been studied in yeast (Table 2). In order to investigate the pathogenic effect of variants identified by whole exome sequencing in TRAPPC4, Van Bergen et al. used the temperature-sensitive *S. cerevisiae* trs23 ts mutant strain [63]. *TRS23* is the yeast ortholog of TRAPPC4, sharing 45% of homology. In this mutant, both the levels of the Trs23 protein and the full TRAPP complex are low. Moreover, at restrictive temperatures, the yeast *trs23ts* strain displayed autophagy and secretion defects similar to that observed in patient fibroblasts. Following these observations, they demonstrated that yeast defect could be complemented by reintroduction of the wt *TRAPPC4* gene in a rescue plasmid; on the other hand, this does not occur with the variants identified in the families because they maintain autophagy and secretion defect due to a low level of the TRAPP subunit. The results obtained in yeast resulted in the conclusion that the severe neurological disorders observed in patients with new TRAPPC4 variants could be caused by the autophagy defect [63,64].

A classical complementation assay has been performed to determine the impact of 36 variants of *ATP7A* gene found in 36 patients with Menkes disease, a congenital neurodegenerative disorder. *ATP7A* encodes a copper-transporting P-type ATPase, which exhibits copper-dependent trafficking. ATP7A is found in the Trans-Golgi Network (TGN) at low copper concentrations and in the post-Golgi compartments, and the plasma membrane at higher concentrations. A hypothesis formulated on how the mutations could affect the transporter has been verified in the *ccc2Δ* yeast strain, deleted for the yeast ortholog of *ATP7A* (Table 2). The *ccc2Δ* yeast strain is unable to grow under iron-limited conditions, but the expression of wild type ATP7A can rescue the growth. Thanks to a spot assay in medium in the presence or in the absence of copper or growth in liquid medium in the presence of several copper concentrations, the authors could determine the difference in functionality of TNG- trapped variants and post-TGN variants [65].

### 4.2. Yeast Assays for Genes Involved in Cancer

Cancer is a complex disease that involves uncontrolled proliferation of cells with the ability to spread to other parts of the body. Mutations in oncogenes or oncosuppressors are frequent in cancers and, especially for highly polymorphic genes, it is extremely important for determining the impact of variants on the function of proteins. Yeast has been largely employed to study oncogenes and oncosuppressors. It has been largely used to determine the functional impact of VUS in p53, a transcription factor frequently mutated in cancer, in PTEN and oncosuppressor BRCA1 [11,66,67,68,69,70,71,72,73,74].

An oncogene that has been studied in yeast is the human *BRAF* (Table 3), a Ser-Thr kinase that belongs to the highly oncogenic RAS/RAF/MEK/ERK (MAPK) signaling pathway found mutated in the kinase domain (mutation V600E) in several cancers [75]. This mutation results in constitutive activation of BRAF kinase function. The BRAFD594V mutation, identified less frequently in cancer known as kinase-dead mutation, has reduced kinase activity [76]. *BRAF* has no ortholog in yeast, but the MAPK pathway is highly conserved. It has been demonstrated that, in yeast, wt BRAF does not complement the activity of kinase at same level in MAPK pathway; interestingly, the mutated BRAFV600E is able to rescue the growth in high osmolarity conditions of the triple mutant *ste11∆ssk2∆ssk22∆* (Table 4). Moreover, the mutation D594V resulted inactive as wt BRAF. By means of a cDNA library screening, a new interactor of BRAFV600E was identified and validated in melanoma cells [19].

The tumor suppressor gene *CHEK2* has also been modeled in the yeast *S. cerevisiae*. *CHEK2* encodes a serine-threonine kinase, which plays an important role in genomic integrity and cellular response to DNA damage through phosphorylation of substrates such as, p53 [77], Cdc25A [78] and Cdc25C phosphatases [79], E2F-1 transcription factor [80], and promyelocytic leukemia protein [81]. *CHEK2* is of great interest in cancer research mainly because of the large number of VUS identified, as well as because of the association of *CHEK2* pathogenic variants alleles with an increased risk of various types of cancers [82]. Delimitsou et al. [83] evaluated the CHEK2 VUS with a yeast-based functional assay. The authors studied 120 germline *CHEK2* missense variants selected from genetic test results in breast cancer families or from the ClinVar database. The yeast ortholog of *CHEK2* is *RAD53*. The *rad53∆* strain is viable and sensitive to DNA damaging agents such as MMS (Table 4). This sensitivity is complemented by the expression of CHEK2. Therefore, the assay for studying CHEK2 VUS has been performed by testing viability in the presence of MMS. This assay in combination with prediction models and family history of breast cancer patients carrying *CHEK2* missense variants enabled the authors to categorize the variants in three distinct groups, demonstrating that the results of the yeast-based assay can complement and facilitate the classification of relatively rare *CHEK2* VUS [83]. 

In addition to the mutations of genes involved in cell cycle and growth, cancer cells also carry mutations in genes involved in metabolism. This is the case of succinate dehydrogenase (SDH), a complex of four-subunit with mitochondrial localization encoded by nuclear genes (*SDHA*, *SDHB*, *SDHC*, and *SDHD*, collectively referred to as *SDHx*). The assembled SDH complex is involved in two essential energy-producing metabolic processes of the cell, the Krebs cycle and the electron transport chain [84]. Loss-of-function mutations affecting the SDH complex predispose patients to develop multiple cancers, called SDH-deficient neoplasia [85]. Tumor formation occurs following the complete loss of function of at least one SDHx subunit. In a study by Bannon et al. [86], data from clinical observations, a functional yeast-based assay, and from a computational analysis were combined to determine the pathogenicity of 22 SDHA VUS. The yeast assay for identifying the functional effect of a VUS was based on *sdh1Δ* (ortholog of *SDHA*) that do not have a functional SDH complex, and thus non-functional mitochondria. The *sdh1∆* mutant is able to grow in the presence of a fermentable carbon source (glucose) but not in the presence of a non-fermentable carbon source (glycerol). Since the expression of the wt Sdh1 restores the growth on glycerol, this assay was the readout for measuring the activity of *SDHA* variants. In order to further corroborate the results of variants functionality from growth assays, they tested the functionality of mitochondria. By means of yeast assays and computational model of protein structure of variants, they were able to draw conclusions on functional impact of variants [86]. 

## 5. Limitations of the Yeast Model

The use of yeast has, of course, some limitations. We have to consider that some human proteins expressed in the model organism will not be as functional as in their native context of a human cell. This can be determined by the fact that some variants may alter interactions with proteins not present or too divergent in yeast; some may impact protein expression/activity by altering transcript splicing or binding sites for miRNA or mRNA binding proteins, which are conditions that cannot be recreated in yeast expressing the cDNA. Moreover, as a unicellular model organism, yeast fails to capture phenotypes resulting from inter-cellular interactions so it may not be suitable, for instance, for studying genes involved in development [87]. However, many of these genes have some conserved functions in yeast, and yeast can be still used to study fundamental biological processes as most genes ultimately function in individual cells. 

## 6. Conclusions

We showed here a significant selection of assays developed in the yeast S. cerevisiae for understanding human genetic variation. Moreover, we showed that yeast assays can be developed and further validated for studying human proteins with different functions and involved in several pathways and diseases. A precondition for setting functional assays is the identification of a measurable phenotype and, as shown here, yeast is a simple and tractable organism, sharing conserved pathways with higher eukaryotes; this allows facilitating the identification of the experimental conditions in order to study a specific protein. An added value is the availability of tools such as strain collections, databases, molecular toolkits and methods that have accumulated over the years [88,89,90]. The potentiality of humanized yeast goes beyond the functional assays discussed here. Yeast has also been exploited to study mutations that are not yet identified in patients, especially designed to gain information about enzyme activity and interaction with the substrate. For example, yeast has been successfully used to examine the relationship between the ZMPSTE24 enzyme and Laminin A substrate involved in Hutchinson–Gilford progeria syndrome (HGPS) [91,92] or to investigate how the interaction ligand/receptor can be improved for a more efficacious therapy, as conducted for IL-10 and IL-10Rβ receptors by yeast surface display engineering platforms [93].

The results from yeast functional assays cannot stand alone; hence, they are considered as indications complementary to additional clinical and experimental data. Yeast functional assays can be also considered as a method for rationally prioritizing experiments to be conducted in more complicated and expensive model organisms [12,94]. In a perspective of increasing knowledge of genome variability, the use of model organisms has become of pivotal importance. 

## Figures and Tables

**Table 1:** List of genes involved in Mendelian diseases that were studied in the yeast *Saccharomyces cerevisiae*.

Name of the Human Gene	Molecular Function	Biological Process	Disease
Cystathionine β-synthase (*CBS*)	Cystathionine β-synthase activity. Hydro-lyase catalyzing the first step of the trans sulfuration pathway.	Cysteine biosynthetic process	Cystathionine β-synthase deficiency
Phosphoserine aminotransferase (*PSAT1*)	Pyridoxal phosphate binding.	L-serine biosynthetic process	Phosphoserine aminotransferase deficiency (PSATD); Neu-Laxova syndrome 2 (NLS2)
Superoxide dismutase (*SOD1*)	Superoxide dismutase activity. Destroys radicals which are normally produced within the cells.	Cellular response to oxidative stress	Familiar amyotrophic lateral sclerosis 1 (fALS1); Spastic tetraplegia and axial hypotonia, progressive (STAHP)
Adenine phosphoribosyl transferase (*APRT*)	Transferase activity and adenine binding. Catalyzes a salvage reaction resulting in the formation of AMP.	Nucleoside metabolic process	Adenine phosphoribosyltransferase deficiency (APRTD)
Triokinase/FMN cyclase (*TKFC*)	Kinase activity on dihydroxyacetone and of glyceraldehydes. It catalyzes the splitting of ribonucleoside diphosphate-X compounds.	Cellular carbohydrate metabolic process	Triokinase and FMN cyclase deficiency syndrome (TKFCD)
Methionine-tRNA synthetase (*MARS1*)	Methionine-tRNA ligase activity. Catalyzes the ATP-dependent ligation of methionine to the 3′-end of its cognate tRNA.	tRNA aminoacylation for protein translation	Charcot-Marie-Tooth disease 2U (CMT2U); interstitial lung and liver disease
Glycyl-tRNA synthetase (*GARS1*)	Glycyl-tRNA ligase activity. Catalyzes the ATP-dependent ligation of glycine to the 3′-end of its cognate tRNA.	tRNA aminoacylation for protein translation	Charcot-Marie-Tooth disease 2D (CMT2D); infantile James type (SMAJI); neuronopathy distal hereditary motor (HMN5A).
Valyl-tRNAsynthetase (*VARS1*)	Valine-tRNA ligase activity. Catalyzes the ATP-dependent ligation of glycine to the 3′-end of its cognate tRNA.	tRNA aminoacylation for protein translation	Neurodevelopmental disorder with microcephaly, seizures, and cortical atrophy (NDMSCA)
DNA-directed RNA polymerase II subunit RPB1 (*POLR2A*)	DNA-directed 5′-3′ RNA polymerase activity.	RNA metabolic process	Neurodevelopmental disorder with hypotonia and variable intellectual and behavioral abnormalities (NEDHIB)
Glutamine-dependent NAD (+) synthetase (*NADSYN1*)	NAD+ synthase (glutamine-hydrolyzing) activity.	de novo NAD biosyntheticprocess	NAD deficiency disorder; Vertebral, cardiac, renal, and limb defects syndrome 3 (VCRL3)
Pyridine nucleotide-disulfide oxidoreductase domain-containing protein 1(*PYROXD1*)	Superoxide dismutase activity. Destroys radicals which are normally produced within the cells.	Cellular response to oxidative stress	Myofibrillar myopathy-8 (MFM8)
Trafficking protein particle complex subunit 2-like protein (*TRAPPC2L*)	Core component of the TRAPP complexes. Targeting and fusion of endoplasmic reticulum-to-Golgi transport vesicles.	Endoplasmic reticulum to Golgi vesicle-mediated transport	Encephalopathy, progressive, early-onset, with episodic rhabdomyolysis (PEERB)
Trafficking protein particle complex subunit 4 (*TRAPPC4*)	Core component of the TRAPP complexes. Targeting and fusion of endoplasmic reticulum-to-Golgi transport vesicles.	Endoplasmic reticulum to Golgi vesicle-mediated transport	Neurodevelopmental disorder with epilepsy, spasticity, and brain atrophy (NEDESBA)
Copper-transporting ATPase 1 (*ATP7A*)	Copper ion transmembrane transporter activity.	Cellular copper ion homeostasis	Menkes disease (MNK); Occipital horn syndrome (OHS); Distal spinal muscular atrophy, X-linked, 3 (DSMAX3)

**Table 2:** Functional assays of genes listed in Table 1 and described in the text.

Human Gene	Yeast Ortholog	Essentiality	Phenotype
*CBS*	*CYS4*	Non-essential	Auxotrophic for cysteine, glutathione, vitamin B6
*PSAT1*	*SER1*	Non-essential	Auxotrophic for serine
*SOD1*	*SOD1*	Non-essential	Growth curve, expression level of mutants; mitochondrial oxygen consumption
*APRT*	*APT1, APT2*	Non-essential	Auxotrophic for purine in the triple mutant *ade2∆ apt1∆ aah1∆*
*TKFC*	*DAK1, DAK2*	Non-essential	Ability to grow in dihydroxyacetone (DHA)
*MARS1*	*MES1*	Essential	Growth defect
*GARS1*	*GRS1*	Essential	Growth defect
*VARS1*	*VAS1*	Essential	Growth defect
*POLR2A*	*RPB1*	Essential	Growth defect
*NADSYN1*	*QNS1*	Essential	Auxotrophic for nicotinamide riboside (NR)
*PYROXD1*	No ortholog, gene with similar activity	-	Growth in the presence of H_2_O_2_
*TRAPPC2L*	*TCA17*	Non-essential	yeast strain ts *tca17∆TRS130-HA*
*TRAPPC4*	*TRS23*	Essential	Trs23*ts*
*ATP7A*	*CCC2*	Non-essential	Growth under iron limited condition

**Table 3:** List of genes involved in cancer that were studied in the yeast, *S. cerevisiae*, described in this review.

Name of the Human Gene	Molecular Function	Biological Process	Disease
*BRAF*	Serine-threonin kinase	Activation of MAPKK activity (RAS/RAF/MEK/ERK kinase pathway)	Melanoma, Papillary thyroid carcinoma, colon rectal cancer, hairy cell leukemia
*CHEK2*	Serine-threonin kinase	DNA damage checkpoint	Li-Fraumeni syndrome 2, breast cancer
*SDHA*	Electron transfer activity; succinate dehydrogenase (ubiquinone) activity	Mitochondrial electron transport, succinate to ubiquinone	SDH-deficient neoplasia, gastrointestinal stromal tuomor (GIST)

**Table 4:** Functional assays of genes listed in Table 2 and described in the text.

Human Gene	Yeast Ortholog	Essentiality	Phenotype
*BRAF*	No ortholog	-	Growth in high concentration of NaCl, in the strain *ste11∆ssk2∆ssk22∆*
*CHEK2*	*RAD53*	Non-essential	Growth in the presence of MMS
*SDHA*	*SDH1*	Non-essential	Growth in non-fermentable carbon source

## Data Availability

Data sharing not applicable.

## References

[1] Lupski J.R., Belmont J.W., Boerwinkle E., Gibbs R.A. (2011). Clan Genomics and the Complex Architecture of Human Disease. Cell.

[2] Zhao F., Zheng L., Goncearenco A., Panchenko A.R., Li M. (2018). Computational Approaches to Prioritize Cancer Driver Missense Mutations. Int. J. Mol. Sci..

[3] Richards S., Aziz N., Bale S., Bick D., Das S., Gastier-Foster J., Grody W.W., Hegde M., Lyon E., Spector E. (2015). Standards and guidelines for the interpretation of sequence variants: A joint consensus recommendation of the American College of Medical Genetics and Genomics and the Association for Molecular Pathology. Genet. Med..

[4] Castellana S., Mazza T. (2013). Congruency in the prediction of pathogenic missense mutations: State-of-the-art web-based tools. Brief. Bioinform..

[5] Frousios K., Iliopoulos C.S., Schlitt T., Simpson M.A. (2013). Predicting the functional consequences of non-synonymous DNA sequence variants—Evaluation of bioinformatics tools and development of a consensus strategy. Genomics.

[6] Gnad F., Baucom A., Mukhyala K., Manning G., Zhang Z. (2013). Assessment of computational methods for predicting the effects of missense mutations in human cancers. BMC Genom..

[7] Boycott K.M., Campeau P., Howley H.E., Pavlidis P., Rogic S., Oriel C., Berman J.N., Hamilton R.M., Hicks G., Lipshitz H.D. (2020). The Canadian Rare Diseases Models and Mechanisms (RDMM) Network: Connecting Understudied Genes to Model Organisms. Am. J. Hum. Genet..

[8] Mizushima N. (2017). The exponential growth of autophagy-related research: From the humble yeast to the Nobel Prize. FEBS Lett..

[9] Giaever G., Nislow C. (2014). The Yeast Deletion Collection: A Decade of Functional Genomics. Genetics.

[10] Wangler M.F., Yamamoto S., Chao H.-T., Posey J.E., Westerfield M., Postlethwait J., Hieter P., Boycott K.M., Campeau P.M., Bellen H.J. (2017). Model Organisms Facilitate Rare Disease Diagnosis and Therapeutic Research. Genetics.

[11] Cervelli T., Lodovichi S., Bellè F., Galli A. (2020). Yeast-based assays for the functional characterization of cancer-associated variants of human DNA repair genes. Microb. Cell.

[12] Dunham M.J., Fowler D.M. (2013). Contemporary, yeast-based approaches to understanding human genetic variation. Curr. Opin. Genet. Dev..

[13] Laurent J.M., Young J.H., Kachroo A.H., Marcotte E.M. (2016). Efforts to make and apply humanized yeast. Brief. Funct. Genom..

[14] Manon S. (2018). Investigating BCL-2 Family Protein Interactions in Yeast. Adv. Struct. Saf. Stud..

[15] La Ferla M., Mercatanti A., Rocchi G., Lodovichi S., Cervelli T., Pignata L., Caligo M., Galli A. (2015). Expression of human poly (ADP-ribose) polymerase 1 in Saccharomyces cerevisiae: Effect on survival, homologous recombination and identification of genes involved in intracellular localization. Mutat. Res. Mol. Mech. Mutagen..

[16] Millot G.A., Berger A., Lejour V., Boulé J.-B., Bobo C., Cullin C., Lopes J., Stoppa-Lyonnet D., Nicolas A. (2011). Assessment of human nter and cterBRCA1mutations using growth and localization assays in yeast. Hum. Mutat..

[17] Inga A., Resnick M. (2001). Novel human p53 mutations that are toxic to yeast can enhance transactivation of specific promoters and reactivate tumor p53 mutants. Oncogene.

[18] Tenreiro S., Franssens V., Winderickx J., Outeiro T.F. (2017). Yeast models of Parkinson’s disease-associated molecular pathologies. Curr. Opin. Genet. Dev..

[19] Lubrano S., Comelli L., Piccirilli C., Marranci A., Dapporto F., Tantillo E., Gemignani F., Gutkind J.S., Salvetti A., Chiorino G. (2019). Development of a yeast-based system to identify new hBRAFV600E functional interactors. Oncogene.

[20] Mercatanti A., Lodovichi S., Cervelli T., Galli A. (2017). CRIMEtoYHU: A new web tool to develop yeast-based functional assays for characterizing cancer-associated missense variants. FEMS Yeast Res..

[21] Guimier A., Gordon C.T., Godard F., Ravenscroft G., Oufadem M., Vasnier C., Rambaud C., Nitschke P., Bole-Feysot C., Masson C. (2016). Biallelic PPA2 Mutations Cause Sudden Unexpected Cardiac Arrest in Infancy. Am. J. Hum. Genet..

[22] Agmon N., Temple J., Tang Z., Schraink T., Baron M., Chen J., Mita P., A Martin J., Tu B.P., Yanai I. (2020). Phylogenetic debugging of a complete human biosynthetic pathway transplanted into yeast. Nucleic Acids Res..

[23] Lee M.-S., Yu M., Kim K.-Y., Park G.-H., Kwack K., Kim K.P. (2015). Functional Validation of Rare Human Genetic Variants Involved in Homologous Recombination Using Saccharomyces cerevisiae. PLoS ONE.

[24] Kim C., Yang J., Jeong S.-H., Kim H., Park G.-H., Shin H.B., Ro M., Kim K.-Y., Park Y., Kim K.P. (2018). Yeast-based assays for characterization of the functional effects of single nucleotide polymorphisms in human DNA repair genes. PLoS ONE.

[25] Greene A.L., Snipe J.R., Gordenin D.A., Resnick M.A. (1999). Functional Analysis of Human FEN1 in Saccharomyces Cerevisiae and Its Role in Genome Stability. Hum. Mol. Genet..

[26] Sun S., Yang F., Tan G., Costanzo M., Oughtred R., Hirschman J., Theesfeld C.L., Bansal P., Sahni N., Yi S. (2016). An extended set of yeast-based functional assays accurately identifies human disease mutations. Genome Res..

[27] Nottingham R.M., Ganley I., Barr F., Lambright D.G., Pfeffer S.R. (2011). RUTBC1 Protein, a Rab9A Effector That Activates GTP Hydrolysis by Rab32 and Rab33B Proteins. J. Biol. Chem..

[28] Yang F., Sun S., Tan G., Costanzo M., Hill D.E., Vidal M., Andrews B.J., Boone C., Roth F.P. (2017). Identifying pathogenicity of human variants via paralog-based yeast complementation. PLoS Genet..

[29] Hamza A., Tammpere E., Kofoed M., Keong C., Chiang J., Giaever G., Nislow C., Hieter P. (2015). Complementation of Yeast Genes with Human Genes as an Experimental Platform for Functional Testing of Human Genetic Variants. Genetics.

[30] Tong A.H.Y., Boone C. (2006). Synthetic Genetic Array Analysis in Saccharomyces cerevisiae. Yeast Protoc..

[31] Kuzmin E., Sharifpoor S., Baryshnikova A., Costanzo M., Myers C.L., Andrews B.J., Boone C. (2014). Synthetic Genetic Array Analysis for Global Mapping of Genetic Networks in Yeast. Methods Mol. Biol..

[32] Young B.P., Post K.L., Chao J.T., Meili F., Haas K., Loewen C.J.R. (2020). Sentinel Interaction Mapping (SIM)—A generic approach for the functional analysis of human disease gene variants using yeast. Dis. Model. Mech..

[33] Young B.P., Loewen C.J. (2013). Balony: A software package for analysis of data generated by synthetic genetic array experiments. BMC Bioinform..

[34] Post K.L., Belmadani M., Ganguly P., Meili F., Dingwall R., McDiarmid T.A., Meyers W.M., Herrington C., Young B.P., Callaghan D.B. (2020). Multi-model functionalization of disease-associated PTEN missense mutations identifies multiple molecular mechanisms underlying protein dysfunction. Nat. Commun..

[35] Gerritsen T., Vaughn J.G., Waisman H.A. (1962). The identification of homocystine in the urine. Biochem. Biophys. Res. Commun..

[36] Ono B., Shirahige Y., Nanjoh A., Andou N., Ohue H., Ishino-Arao Y. (1988). Cysteine biosynthesis in Saccharomyces cerevisiae: Mutation that confers cystathionine beta-synthase deficiency. J. Bacteriol..

[37] Kruger W.D., Cox D.R. (1995). A yeast assay for functional detection of mutations in the human cystathionine -synthase gene. Hum. Mol. Genet..

[38] Mayfield J.A., Davies M.W., Dimster-Denk D., Pleskac N., McCarthy S., Boydston E.A., Fink L., Lin X.X., Narain A.S., Meighan M. (2012). Surrogate Genetics and Metabolic Profiling for Characterization of Human Disease Alleles. Genetics.

[39] Hidalgo R.A., Schanze D., Kariminejad A., Nordgren A., Kariminejad M.H., Conner P., Grigelioniene G., Nilsson D., Nordenskjöld M., Wedell A. (2014). Neu-Laxova Syndrome Is a Heterogeneous Metabolic Disorder Caused by Defects in Enzymes of the L-Serine Biosynthesis Pathway. Am. J. Hum. Genet..

[40] Sirr A., Lo R.S., Cromie G.A., Scott A.C., Ashmead J., Heyesus M., Dudley A.M. (2020). A yeast-based complementation assay elucidates the functional impact of 200 missense variants in human PSAT1. J. Inherit. Metab. Dis..

[41] Bastow E.L., Peswani A.R., Tarrant D.S.J., Pentland D.R., Chen X., Morgan A., Staniforth G.L., Tullet J.M., Rowe M.L., Howard M. (2016). New links between SOD1 and metabolic dysfunction from a yeast model of Amyotrophic Lateral Sclerosis (ALS). J. Cell Sci..

[42] Bollee G., Harambat J., Bensman A., Knebelmann B., Daudon M., Ceballos-Picot I. (2012). Adenine Phosphoribosyltransferase Deficiency. Clin. J. Am. Soc. Nephrol..

[43] Huyet J., Ozeir M., Burgevin M.-C., Pinson B., Chesney F., Remy J.-M., Siddiqi A.R., Lupoli R., Pinon G., Saint-Marc C. (2018). Structural Insights into the Forward and Reverse Enzymatic Reactions in Human Adenine Phosphoribosyltransferase. Cell Chem. Biol..

[44] Rodrigues J.R., Couto A., Cabezas A., Pinto R.M., Ribeiro J., Canales J., Costas M.J., Cameselle J.C. (2014). Bifunctional Homodimeric Triokinase/FMN Cyclase. J. Biol. Chem..

[45] Wortmann S.B., Meunier B., Mestek-Boukhibar L., Broek F.V.D., Maldonado E.M., Clement E., Weghuber D., Spenger J., Jaros Z., Taha F. (2020). Bi-allelic Variants in TKFC Encoding Triokinase/FMN Cyclase Are Associated with Cataracts and Multisystem Disease. Am. J. Hum. Genet..

[46] Gillespie M.K., McMillan H.J., Kernohan K.D., Pena I.A., Meyer-Schuman R., Antonellis A., Boycott K.M. (2019). A Novel Mutation in MARS in a Patient with Charcot-Marie-Tooth Disease, Axonal, Type 2U with Congenital Onset. J. Neuromuscul. Dis..

[47] Markovitz R., Ghosh R., Kuo M.E., Hong W., Lim J., Bernes S., Manberg S., Crosby K., Tanpaiboon P., Bharucha-Goebel D. (2020). GARS-related disease in infantile spinal muscular atrophy: Implications for diagnosis and treatment. Am. J. Med. Genet. Part A.

[48] Siekierska A., Stamberger H., Deconinck T., Oprescu S.N., Partoens M., Zhang Y., Sourbron J., Adriaenssens E., Mullen P., Wiencek P. (2019). Biallelic VARS variants cause developmental encephalopathy with microcephaly that is recapitulated in vars knockout zebrafish. Nat. Commun..

[49] Gonzalez M., McLaughlin H., Houlden H., Guo M., Yo-Tsen L., Hadjivassilious M., Speziani F., Yang X.-L., Antonellis A., Reilly M.M. (2013). Exome sequencing identifies a significant variant in methionyl-tRNA synthetase (MARS) in a family with late-onset CMT2. J. Neurol. Neurosurg. Psychiatry.

[50] Oprescu S.N., Griffin L.B., Beg A.A., Antonellis A. (2017). Predicting the pathogenicity of aminoacyl-tRNA synthetase mutations. Methods.

[51] Clark V.E., Harmancı A.S., Bai H., Youngblood M.W., Lee T.I., Baranoski J.F., Ercan-Sencicek A.G., Abraham B., Weintraub A.S., Hnisz D. (2016). Recurrent somatic mutations in POLR2A define a distinct subset of meningiomas. Nat. Genet..

[52] Haijes H., Koster M.J., Rehmann H., Li D., Hakonarson H., Cappuccio G., Hancarova M., Lehalle D., Reardon W., Schaefer G.B. (2019). De Novo Heterozygous POLR2A Variants Cause a Neurodevelopmental Syndrome with Profound Infantile-Onset Hypotonia. Am. J. Hum. Genet..

[53] Hansen A.W., Arora P., Khayat M.M., Smith L.J., Lewis A.M., Rossetti L.Z., Jayaseelan J., Cristian I., Haynes D., DiTroia S. (2021). Germline mutation in POLR2A: A heterogeneous, multi-systemic developmental disorder characterized by transcriptional dysregulation. Hum. Genet. Genom. Adv..

[54] Shi H., Enriquez A., Rapadas M., Martin E.M., Wang R., Moreau J.L.M., Lim E., Szot J., Ip E.K., Hughes J.N. (2017). NAD Deficiency, Congenital Malformations, and Niacin Supplementation. N. Engl. J. Med..

[55] Kirkland J.B. (2012). Niacin requirements for genomic stability. Mutat. Res. Mol. Mech. Mutagen..

[56] Nikiforov A., Kulikova V., Ziegler M. (2015). The human NAD metabolome: Functions, metabolism and compartmentalization. Crit. Rev. Biochem. Mol. Biol..

[57] Suda Y., Tachikawa H., Yokota A., Nakanishi H., Yamashita N., Miura Y., Takahashi N. (2003). Saccharomyces cerevisiae QNS1 codes for NAD+ synthetase that is functionally conserved in mammals. Yeast.

[58] Szot J., Campagnolo C., Cao Y., Iyer K.R., Cuny H., Drysdale T., Flores-Daboub J.A., Bi W., Westerfield L., Liu P. (2020). Bi-allelic Mutations in NADSYN1 Cause Multiple Organ Defects and Expand the Genotypic Spectrum of Congenital NAD Deficiency Disorders. Am. J. Hum. Genet..

[59] O’Grady G.L., Best H.A., Sztal T., Schartner V., Sanjuan-Vazquez M., Donkervoort S., Neto O.L.A., Sutton R.B., Ilkovski B., Romero N.B. (2016). Variants in the Oxidoreductase PYROXD1 Cause Early-Onset Myopathy with Internalized Nuclei and Myofibrillar Disorganization. Am. J. Hum. Genet..

[60] Sainio M., Välipakka S., Rinaldi B., Lapatto H., Paetau A., Ojanen S., Brilhante V., Jokela M., Huovinen S., Auranen M. (2018). Recessive PYROXD1 mutations cause adult-onset limb-girdle-type muscular dystrophy. J. Neurol..

[61] Sacher M., Shahrzad N., Kamel H., Milev M.P. (2019). TRAPPopathies: An emerging set of disorders linked to variations in the genes encoding transport protein particle (TRAPP)-associated proteins. Traffic.

[62] Milev M.P., Graziano C., Karall D., Kuper W.F.E., Al-Deri N., Cordelli D.M., Haack T.B., Danhauser K., Iuso A., Palombo F. (2018). Bi-allelic mutations in TRAPPC2L result in a neurodevelopmental disorder and have an impact on RAB11 in fibroblasts. J. Med. Genet..

[63] Van Bergen N.J., Guo Y., Al-Deri N., Lipatova Z., Stanga D., Zhao S., Murtazina R., Gyurkovska V., Pehlivan D., Mitani T. (2020). Deficiencies in vesicular transport mediated by TRAPPC4 are associated with severe syndromic intellectual disability. Brain.

[64] Lipatova Z., Van Bergen N., Stanga D., Sacher M., Christodoulou J., Segev N. (2020). TRAPPing a neurological disorder: From yeast to humans. Autophagy.

[65] Skjørringe T., Pedersen P.A., Thorborg S.S., Nissen P., Gourdon P., Møller L.B. (2017). Characterization of ATP7A missense mutants suggests a correlation between intracellular trafficking and severity of Menkes disease. Sci. Rep..

[66] Kato S., Han S.-Y., Liu W., Otsuka K., Shibata H., Kanamaru R., Ishioka C. (2003). Understanding the function–structure and function–mutation relationships of p53 tumor suppressor protein by high-resolution missense mutation analysis. Proc. Natl. Acad. Sci. USA.

[67] Carbonnier V., Leroy B., Rosenberg S., Soussi T. (2020). Comprehensive assessment of TP53 loss of function using multiple combinatorial mutagenesis libraries. Sci. Rep..

[68] Monti P., Bosco B., Gomes S., Saraiva L., Fronza G., Inga A. (2019). Yeast As a Chassis for Developing Functional Assays to Study Human P53. J. Vis. Exp..

[69] Andres-Pons A., Rodríguez-Escudero I., Gil A., Blanco A., Vega A., Molina M., Pulido R., Cid V.J. (2007). In vivo Functional Analysis of the Counterbalance of Hyperactive Phosphatidylinositol 3-Kinase p110 Catalytic Oncoproteins by the Tumor Suppressor PTEN. Cancer Res..

[70] Rodríguez-Escudero I., Roelants F.M., Thorner J., Nombela C., Molina M., Cid V.J. (2005). Reconstitution of the mammalian PI3K/PTEN/Akt pathway in yeast. Biochem. J..

[71] Rodríguez-Escudero I., Oliver M.D., Andrés-Pons A., Molina M., Cid V.J., Pulido R. (2011). A comprehensive functional analysis of PTEN mutations: Implications in tumor- and autism-related syndromes. Hum. Mol. Genet..

[72] Monteiro A., August A., Hanafusa H. (1996). Evidence for a transcriptional activation function of BRCA1 C-terminal region. Proc. Natl. Acad. Sci. USA.

[73] Caligo M., Bonatti F., Guidugli L., Aretini P., Galli A. (2009). A yeast recombination assay to characterize humanBRCA1missense variants of unknown pathological significance. Hum. Mutat..

[74] Thouvenot P., Ben Yamin B., Fourriere L., Lescure A., Boudier T., Del Nery E., Chauchereau A., Goldgar D.E., Houdayer C., Stoppa-Lyonnet D. (2016). Functional Assessment of Genetic Variants with Outcomes Adapted to Clinical Decision-Making. PLoS Genet..

[75] Fiskus W., Mitsiades N. (2016). B-Raf Inhibition in the Clinic: Present and Future. Annu. Rev. Med..

[76] Michaloglou C., Vredeveld L.C.W., Mooi W.J., Peeper D.S. (2007). BRAFE600 in benign and malignant human tumours. Oncogene.

[77] Hirao A., Kong Y.-Y., Matsuoka S., Wakeham A., Ruland J., Yoshida H., Liu D., Elledge S.J., Mak T.W. (2000). DNA Damage-Induced Activation of p53 by the Checkpoint Kinase Chk2. Science.

[78] Falck J., Mailand N., Syljuåsen R.G., Bartek J., Lukas J. (2001). The ATM–Chk2–Cdc25A checkpoint pathway guards against radioresistant DNA synthesis. Nature.

[79] Brown A.L., Lee C.-H., Schwarz J.K., Mitiku N., Piwnica-Worms H., Chung J.H. (1999). A human Cds1-related kinase that functions downstream of ATM protein in the cellular response to DNA damage. Proc. Natl. Acad. Sci. USA.

[80] Stevens C., Smith L., La Thangue N.B. (2003). Chk2 activates E2F-1 in response to DNA damage. Nat. Cell Biol..

[81] Yang S., Kuo C., Bisi J.E., Kim M.K. (2002). PML-dependent apoptosis after DNA damage is regulated by the checkpoint kinase hCds1/Chk2. Nat. Cell Biol..

[82] Stolarova L., Kleiblova P., Janatova M., Soukupova J., Zemankova P., Macurek L., Kleibl Z. (2020). *CHEK2* Germline Variants in Cancer Predisposition: Stalemate Rather than Checkmate. Cells.

[83] Delimitsou A., Fostira F., Kalfakakou D., Apostolou P., Konstantopoulou I., Kroupis C., Papavassiliou A.G., Kleibl Z., Stratikos E., Voutsinas G.E. (2019). Functional characterization of CHEK2 variants in a Saccharomyces cerevisiae system. Hum. Mutat..

[84] Rustin P., Munnich A., Rotig A. (2002). Succinate dehydrogenase and human diseases: New insights into a well-known enzyme. Eur. J. Hum. Genet..

[85] Gill A.J. (2017). Succinate dehydrogenase (SDH)-deficient neoplasia. Histopathology.

[86] Bannon A.E., Kent J.D., Forquer I., Town A., Klug L.R., McCann K., Beadling C., Harismendy O., Sicklick J.K., Corless C.L. (2017). Biochemical, Molecular, and Clinical Characterization of Succinate Dehydrogenase Subunit A Variants of Unknown Significance. Clin. Cancer Res..

[87] Mohammadi S., Saberidokht B., Subramaniam S., Grama A. (2015). Scope and limitations of yeast as a model organism for studying human tissue-specific pathways. BMC Syst. Biol..

[88] Franssens V., Bynens T., Brande J.V.D., Vandermeeren K., Verduyckt M., Winderickx J. (2013). The Benefits of Humanized Yeast Models to Study Parkinson’s Disease. Oxidative Med. Cell. Longev..

[89] Hofer S., Kainz K., Zimmermann A., Bauer M.A., Pendl T., Poglitsch M., Madeo F., Carmona-Gutierrez D. (2018). Studying Huntington’s Disease in Yeast: From Mechanisms to Pharmacological Approaches. Front. Mol. Neurosci..

[90] Seynnaeve D., Del Vecchio M., Fruhmann G., Verelst J., Cools M., Beckers J., Mulvihill D.P., Winderickx J., Franssens V. (2018). Recent Insights on Alzheimer’s Disease Originating from Yeast Models. Int. J. Mol. Sci..

[91] Spear E.D., Alford R., Babatz T.D., Wood K.M., Mossberg O.W., Odinammadu K., Shilagardi K., Gray J.J., Michaelis S. (2019). A humanized yeast system to analyze cleavage of prelamin A by ZMPSTE24. Methods.

[92] Babatz T.D., Spear E.D., Xu W., Sun O.L., Nie L., Carpenter E.P., Michaelis S. (2021). Site specificity determinants for prelamin A cleavage by the zinc metalloprotease ZMPSTE24. J. Biol. Chem..

[93] Gorby C., Bellón J.S., Wilmes S., Warda W., Pohler E., Fyfe P.K., Cozzani A., Ferrand C., Walter M.R., Mitra S. (2020). Engineered IL-10 variants elicit potent immunomodulatory effects at low ligand doses. Sci. Signal..

[94] Lehner B. (2013). Genotype to phenotype: Lessons from model organisms for human genetics. Nat. Rev. Genet..

